# Identifying Stride-To-Stride Control Strategies in Human Treadmill Walking

**DOI:** 10.1371/journal.pone.0124879

**Published:** 2015-04-24

**Authors:** Jonathan B. Dingwell, Joseph P. Cusumano

**Affiliations:** 1 Department of Kinesiology & Health Education, University of Texas, Austin, Texas, United States of America; 2 Department of Engineering Science & Mechanics, Pennsylvania State University, University Park, Pennsylvania, United States of America; VU University Amsterdam, NETHERLANDS

## Abstract

Variability is ubiquitous in human movement, arising from internal and external noise, inherent biological redundancy, and from the neurophysiological control actions that help regulate movement fluctuations. Increased walking variability can lead to increased energetic cost and/or increased fall risk. Conversely, biological noise may be beneficial, even necessary, to enhance motor performance. Indeed, encouraging more variability actually facilitates greater improvements in some forms of locomotor rehabilitation. Thus, it is critical to identify the fundamental principles humans use to regulate stride-to-stride fluctuations in walking. This study sought to determine how humans regulate stride-to-stride fluctuations in stepping movements during treadmill walking. We developed computational models based on pre-defined goal functions to compare if subjects, from each stride to the next, tried to maintain the same *speed* as the treadmill, or instead stay in the same *position* on the treadmill. Both strategies predicted average behaviors empirically indistinguishable from each other and from that of humans. These strategies, however, predicted very different stride-to-stride fluctuation dynamics. Comparisons to experimental data showed that human stepping movements were generally well-predicted by the speed-control model, but not by the position-control model. Human subjects also exhibited no indications they corrected deviations in absolute position only intermittently: i.e., closer to the boundaries of the treadmill. Thus, humans clearly do not adopt a control strategy whose primary goal is to maintain some constant absolute position on the treadmill. Instead, humans appear to regulate their stepping movements in a way most consistent with a strategy whose *primary* goal is to try to maintain the same speed as the treadmill at each consecutive stride. These findings have important implications both for understanding how biological systems regulate walking in general and for being able to harness these mechanisms to develop more effective rehabilitation interventions to improve locomotor performance.

## Introduction

When humans or animals walk, they have to perform a very complex task, especially when walking in unpredictable environments [1,2]. The neural systems that regulate walking [3,4] must continually integrate multiple sensory inputs and coordinate motor outputs to numerous actuators to achieve efficient, stable, and adaptable locomotion. Both external disturbances [5,6] and internal physiological noise [7,8] make every step we take slightly different and thus contribute to the variability observed in walking movements. However, the underlying meaning and functional implications of this variability are not clear. On the one hand, increased intrinsic variability of some gait variables predicts an increased fall risk in the elderly [9,10]. Conversely, allowing or even purposefully imposing increased movement variability during robotic gait re-training can facilitate improved locomotor outcomes [11–13] during rehabilitation. So whereas in some contexts, some forms of locomotor variability may be detrimental, in other contexts, variability has been shown to be beneficial. It is therefore critical to understand how the nervous system regulates such fluctuations when performing complex adaptive movements [14,15] like walking [16]. Doing so will yield important insights into the fundamental principles that govern these processes, which can in turn inform the development of more effective rehabilitation interventions.

Fluctuations in repeated human movements arise from external disturbances, but also from the physiological noise and redundancy that are inherent in biological systems. First, there are multiple sensory and motor sources of physiological noise [8,17]. Second, there are many more mechanical degrees of freedom than required to execute a single movement, many more muscles than required to actuate each degree of freedom, and so on. Redundancy makes biological systems inherently under-constrained and this gives rise to equifinality [15], where an infinite number of movement solutions all equally satisfy some task goal [18–20]. In engineering, both noise and redundancy are most often viewed as disruptive elements to be overcome. It is widely believed humans thus seek to find unique solutions that are “optimal” according to some criterion [21,22]. These optimization approaches, however, have mainly focused on predicting *average* behavior, not on explaining the variability ubiquitously observed in movements like walking [23,24]. Conversely, mounting evidence suggests that inherent biological noise may benefit function, and in some instances may even be necessary to achieve optimal function [14,25–27]. Likewise, redundancy and equifinality may help facilitate adaptability in motor performance [15,19,28,29]. Thus, understanding the interplay between variability, redundancy, and task performance is critical to understanding how humans perform skilled movements.

When humans walk, they need to adapt at *every* step (not just on average) to respond to externally and/or internally generated perturbations [30]. Both these perturbations themselves and the neuro-mechanical responses to them contribute to stride-to-stride variability observed in our movements [23,31]. Thus, the dynamical fluctuations observed in locomotor behavior likely reflect more than just simple random noise [30,32] and changes in the nature of these fluctuations can provide insights into neuromuscular control [31,33]. In particular, measures of variability that average deviations over many cycles cannot capture the corrective responses made from each cycle to the next. In computational models where the variables being controlled and the degree to which they are controlled (e.g., via gains) are specified *a priori*, imposing stochastically optimal control of a particular variable leads to uncorrelated fluctuations in the time series of that variable [16,20,34]. Conversely, *under*-correcting for deviations in a given variable leads to statistical persistence, whereas *over*-correcting leads to statistical anti-persistence [16,20,34]. Thus, differences in the correlation structure between different system outputs can yield direct insights into the degree to which each variable is controlled, independent of the variability exhibited by those variables.

During unconstrained overground walking, fluctuations in stride length (*L*_*n*_), time (*T*_*n*_), and speed (*S*_*n*_) all exhibit strong statistical persistence [35], indicating relatively weak control [16,34] of all three parameters. When those same subjects walked in time with a metronome, their *T*_*n*_ became anti-persistent, but their *L*_*n*_ and *S*_*n*_ remained strongly persistent [35], indicating strong control over only *T*_*n*_, as expected. When walking on a treadmill at fixed speed, humans exhibit slight anti-persistence of stride speed (*S*_*n*_), but strong persistence for both *L*_*n*_ and *T*_*n*_ [16]. However, when walking on a treadmill in time with a metronome, humans tightly regulate all three primary gait variables [36], consistent with the way in which these variables are coupled (i.e., *S*_*n*_ = *L*_*n*_/*T*_*n*_) [16]. These experimental observations are all consistent with our computational framework and with our interpretation of how the fluctuation dynamics of these time series are related to control [16,20,34]. However, they do not by themselves directly rule out other possible alternatives.

While experiments are critical, conclusions drawn from experimental observations alone are often difficult to interpret in terms of underlying physiological processes. Deciphering the origins and implications of stride-to-stride fluctuations in human walking requires a comprehensive computational framework that can generate concrete, experimentally testable *a priori* hypotheses [20]. Building on just such a framework [14,15], we previously formulated goal functions [20] to give concrete mathematical form to hypotheses regarding the strategies humans might use to walk on a motor-driven treadmill [16]. Here, we extend that work by directly testing competing *a priori* hypotheses. In particular, in the sagittal plane, walking on a motor-driven treadmill only requires that subjects not “walk off” either its front or back end. Thus, over time, subjects must stay in the same average position (i.e., roughly “in the middle” of the treadmill), and must walk at the same average speed as the treadmill [16]. However, substantial fluctuations in both position and walking speed arise due to stride-to-stride changes in stride lengths and/or times, and these fluctuations can be sustained over multiple consecutive strides [16,23]. The question addressed here is how do people *regulate* these variations from each stride to the next?

One possible strategy humans could try is to match, at each stride, the same constant *speed* as the treadmill [16]. A distinct alternative strategy that has not previously been carefully considered is to try, at each stride, to remain in the same absolute *position* on the treadmill. Clearly, if the treadmill is operating at constant speed, then over time, trying to walk at the same speed as the treadmill will necessarily result in remaining in the same average absolute position. Likewise, trying to stay in the same position on the treadmill will necessarily result in walking at the same average speed as the treadmill. Thus, it is not at all clear how, or even if, these two strategies are fundamentally different. Indeed, traditional optimal control approaches will predict the exact same *average* movement behavior for both strategies and will therefore be inherently unable to distinguish these alternatives. Here, we hypothesize that at the level of stride-to-stride fluctuations, where control is actually exerted by humans, these two strategies are in fact quite different. We embed these two different strategies in a computational modeling framework that allows us to explicitly predict, *a priori*, what stride-to-stride fluctuation dynamics should result in each case. We then tested those predictions against experimental data from humans.

## Materials and Methods

### Treadmill Walking Defined

Fundamentally, the task of walking is about moving through one’s environment. To achieve forward motion, a bipedal walker (e.g., human, robot, etc.) must move a finite distance in a finite time at each consecutive stride. Thus, the most fundamental and natural observable quantities for walking are stride length (*L*_*n*_) and stride time (*T*_*n*_) at each stride *n*, together with the absolute position (*P*_*n*_) they generate. When walking on a motor-driven treadmill operating at constant speed *v*, the walker’s displacement (i.e., change in position) relative to the inertial laboratory reference frame at each stride is Δ*P*_*n*_ *= L*_*n*_—*vT*_*n*_. Thus, one can typically reduce the description of walking to two independent observables, such as [*T*_*n*_, *L*_*n*_] [16]. Equivalent descriptions of walking dynamics using similar and/or related variables have been used for many years [37,38]. From the perspective of dynamical systems theory, these fundamental stride-to-stride observables arise as a projection of the full neuro-biomechanical system into the [*T*_*n*_, *L*_*n*_] plane of an impact Poincaré section [39] defined by consecutive heel strikes [16].

**Fig 1:**
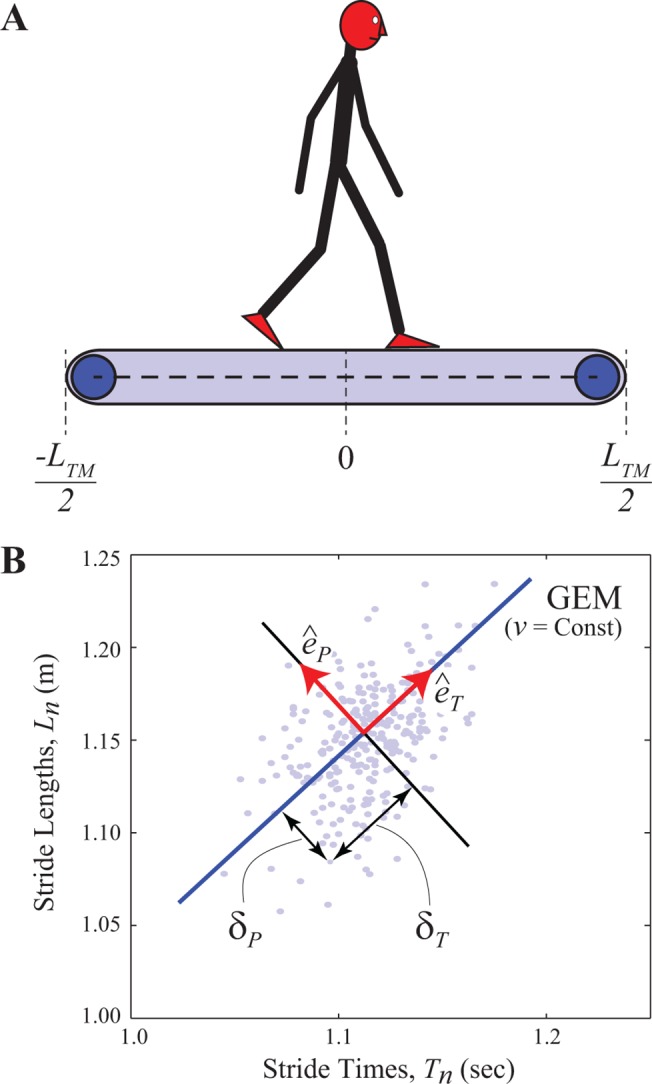
Regulating Stride Length (*L*_*n*_) and Stride Time (*T*_*n*_) When Walking on a Treadmill. (A) Schematic figure of a person walking on a treadmill of total length *L*_***TM***_ with the center position defined as zero. The only strict *requirement* of the task is that the person not walk off either the front (+*L*_***TM***_/2) or back (−*L*_***TM***_ /2) end of the treadmill (Eq 1). (B) Example data for stride times (*T*_***n***_) and stride lengths (*L*_***n***_) for a typical trial for a typical subject. Each dot represents the particular [*T*_***n***_, *L*_***n***_] combination for one individual stride, *n*. The solid diagonal line indicates the set of all combinations of *L*_***n***_ and *T*_***n***_ that achieve the exact same speed, *v*. This line defines one possible Goal Equivalent Manifold (GEM) for walking (Eq 2): i.e., for walking while trying to achieve the goal of maintaining constant speed. Unit vectors then define directions perpendicular to (ê_***P***_) and tangent to (ê_***T***_) the GEM. Deviations *δ*_***P***_ and *δ*_***T***_ define the deviations of each data point in the ê_***P***_ and ê_***T***_ directions, respectively (see Ref. [16]).

For sagittal plane motion (i.e., forward progression), the primary requirement for walking on any treadmill is to simply not walk off of the belt. That is, assuming (without loss of generality) that the walker starts at the center of the treadmill (i.e., *P*_*0*_ = 0), at each subsequent consecutive stride, *n*, we require:

$$\left|\sum_{k=1}^{n}\Delta P_{k}\right|\equiv \left|\sum_{k=1}^{n}\left(L_{k}-vT_{k}\right)\right|<\frac{L_{TM}}{2},$$ (1)

where *L*_*TM*_ is the length of the treadmill belt whose midpoint position is zero (Fig 1A). This summation of all displacements across all previous consecutive strides thus defines the walker’s current absolute position at stride *n* and must be satisfied for all *n* ∊ {1,…,*N*} strides walked.

### Goal-Equivalent Strategies for Treadmill Walking

A key observation is that any sequence of *L*_*n*_ and *T*_*n*_ that satisfies Eq (1) will successfully accomplish the treadmill walking task. Many possible strategies for generating such a sequence exist [16]. Here, we consider two simple and intuitive candidate strategies, which we formulate as goal functions [15], *F*(*T*_*n*_,*L*_*n*_). First, one could choose *L*_*n*_ and *T*_*n*_ at each step to try to maintain the same constant *speed* as the treadmill. This strategy yields the following goal function:

$$F_{Spd}=\frac{L_{n}}{T_{n}}-v.$$ (2)

Alternatively, one could choose *L*_*n*_ and *T*_*n*_ at each step to try to maintain some constant absolute *position* on the treadmill. Assuming (without loss of generality) the desired position to be maintained is at the origin (i.e., 0 in Fig 1A), this yields the following goal function:

$$F_{Pos}=P_{n}=\sum_{k=1}^{n}(\Delta P_{k})=\sum_{k=1}^{n}\left(L_{k}-vT_{k}\right).$$ (3)

Defining treadmill walking this way makes it clear that task performance is completely determined by the observables *L*_*n*_ and *T*_*n*_, independent of the detailed dynamics for all other neuromotor and/or biomechanical degrees of freedom. Upon choosing either strategy, the walker’s task is then to drive the corresponding goal function as close to zero as possible, given inherent system noise. Any value of *F*(*T*_*n*_,*L*_*n*_) ≠ 0 represents a deviation from perfect performance for either goal. In both cases, the set of all possible combinations of [*T*_*n*_, *L*_*n*_] for which *F*(*T*_*n*_,*L*_*n*_) = 0 defines the corresponding Goal Equivalent Manifold (GEM) [15] for that strategy (e.g., Fig 1B). Critically, neither goal function represents a “constraint” on the dynamics, because neither condition represented by Eq (2) or (3) is *required* by Eq (1). Instead, these represent merely two of *many* possible movement strategies that could exist [16], including a variety of possible stochastic (i.e. “drunken”) gaits.

Importantly, the long-time average behaviors of these two candidate movement strategies are empirically indistinguishable. Also, each hypothesized GEM exists prior to, and independent of, any specific control policy people might adopt to regulate their stepping movements [15,16]. Thus, the question then becomes: in what way(s), if any, are these two strategies dynamically distinct? Answering this question requires predicting the stride-to-stride fluctuation dynamics that should arise from implementing either candidate walking strategy using a suitable controller.

### Modelling Stride-to-Stride Regulation

We developed specific stochastic control models that directly implemented each strategy described above. Our intent was to develop phenomenological models that are as simple as possible, but yet still capture the key relevant features of stride-to-stride dynamics. The process for regulating stride-to-stride walking dynamics on the treadmill was modeled as a discrete map, written in the general form of an update equation:

$$x_{n+1}=x_{n}+\text{G}\left(\text{I}+{\text{N}}_{n}\right)u\left(x_{n}\right)+\eta_{n},$$ (4)

where **u**(**x**_*n*_) is a vector of inputs from an optimal inter-stride controller (to be determined), **x**_*n*_ is a suitable controller state variable for current stride *n*, and **x**_*n*+1_ is the corresponding state for the subsequent stride. Also in the above, I is the identity matrix, G is a diagonal matrix denoting additional gains, used as a convenient means to detune the system away from optimality [16], N_*n*_ is a diagonal multiplicative (i.e., motor output) noise matrix, and **η**_*n*_ is a vector representing sensory, perceptual, motor, or other noise sources arising from unmodeled aspects of the overall complex biodynamics of the system.

Operating at the level of experimental observables, we take the inter-trial controller state variable to be **x**_*n*_ = [*T*_*n*_,*L*_*n*_]. This choice is motivated by the fact that the treadmill walking task is completely specified by conditions on *T*_*n*_ and *L*_*n*_ (Eqs 1–3): any controller will have to correct any value of [*T*_*n*_, *L*_*n*_] that deviates from the GEM: i.e., that does not satisfy *F*(*T*_*n*_,*L*_*n*_) = 0. We can thus think of Eq (4) as the “top level” of a hierarchical controller that makes adjustments between strides. The “bottom level” controller is implicit: it is the *intra*-stride component that acts to generate each *L*_*n*_ and *T*_*n*_ during each stride (e.g., [22,40]). In this sense, the inter-stride controller states [*T*_*n*_, *L*_*n*_] act as parameters specifying boundary conditions that *any* suitable intra-trial controller must match. In the absence of noise (**η**_*n*_ = 0 in Eq 4), additional control is unnecessary (**u**(**x**_*n*_) = 0), and the stride-to-stride dynamics in an impact Poincaré section for the complete system exhibit a fixed point where successive strides simply repeat: **x**_*n*+1_ = **x**_*n*_. Thus, Eq (4) captures the essential notion of a central pattern generator process [41,42] that yields repetitive limit cycle behavior [16,22].

The dimensionality of the intra-stride system is very large, including many more state variables (e.g., [40,42,43]) than needed to capture the passive mechanical aspects of gait [5,22,38,44]. Indeed, the dimension of task-relevant neuromotor and perceptual states could range from the hundreds to billions or more, depending on the scale of description. This very high-dimensional aspect of biological systems is indeed the fundamental endogenous source of the fluctuations observed in human movements. Thus, *L*_*n*_ and *T*_*n*_ are analogous to thermodynamic observables in statistical mechanics [45]: they are “macro-states” that represent the output of a complex system, each specific value of which can arise from a vast number of different “micro-states”. From this perspective, Eq (4) is similar to the discrete Langevin Eq in the study of Brownian motion [45]: it is a low-dimensional model of “macroscopic” dynamics driven by a noise term representing the effect of a vast number of internal, “microscopic” degrees-of-freedom.

### Maintaining Constant Walking Speed

To implement a strategy that tries to maintain constant walking speed at each step, we note Eq 4 can be reduced to [16]:

$$\begin{array}{l} T_{n+1}=T_{n}+g_{1}(1+\sigma_{N1}\varepsilon_{N1})u_{1}+\sigma_{\eta 1}\varepsilon_{\eta 1}\\ L_{n+1}=L_{n}+g_{2}(1+\sigma_{N2}\varepsilon_{N2})u_{2}+\sigma_{\eta 2}\varepsilon_{\eta 2} \end{array},$$ (5)

where the gain and noise matrices from Eq 4 become

$$\text{G}=[\begin{array}{cc} g_{1} & 0\\ 0 & g_{2} \end{array}],\;\text{N}=[\begin{array}{cc} \sigma_{N1}\varepsilon_{N1} & 0\\ 0 & \sigma_{N2}\varepsilon_{N2} \end{array}],\;and\;\eta =\left[\begin{array}{c} \sigma_{\eta 1}\varepsilon_{\eta 1}\\ \sigma_{\eta 2}\varepsilon_{\eta 2} \end{array}\right],$$

in which the *ε* represent independent Gaussian random variables with zero mean and unit variance and the *σ* give the corresponding standard deviations of each. For this model, the error relative to the GEM, which is to be regulated, is taken directly from the relevant goal function (Eq 2):

$$e_{Spd}=\frac{L_{n}}{T_{n}}-v$$ (6)

### Maintaining Constant Absolute Position

To implement a strategy that tries to maintain constant position on the treadmill at each step requires that we add a 3^rd^ state variable to track the absolute position (i.e., net total displacement) on the treadmill:

$$\begin{array}{l} T_{n+1}=T_{n}+g_{1}(1+\sigma_{N1}\varepsilon_{N1})u_{1}+\sigma_{\eta 1}\varepsilon_{\eta 1}\\ L_{n+1}=L_{n}+g_{2}(1+\sigma_{N2}\varepsilon_{N2})u_{2}+\sigma_{\eta 2}\varepsilon_{\eta 2}\\ P_{n+1}=P_{n}+(L_{n+1}-vT_{n+1}) \end{array},$$ (7)

where $P_{n}=\sum_{k=1}^{n}\left(\Delta P_{k}\right)$ is the walker’s current absolute position at stride *n*, equivalently defined as net cumulative distance walked up until that stride. Note that no control is applied directly to *P*_*n*_ itself. While all three controller states are “observable” to human subjects, only *T*_*n*_ and *L*_*n*_ are “controllable”. The error relative to the GEM that is to be regulated for this model is then also taken directly from its relevant goal function (Eq 3):

$$e_{Pos}=P_{n}$$ (8)

### Stochastic Optimal Control

The controller was modeled as an unbiased stochastic optimal single-step controller with direct error feedback, based on the Minimum Intervention Principle (MIP) [14,18], but modified to include the cost of deviating from a “preferred operating point”, [T*, L*], along the GEM [16]. This additional cost is added because perfect MIP control is based only on task error (Eq 6 and 8): it does not consider that human legs have finite length, or inertia, nor that humans adopt a combination of stride length and stride time that tends to minimize energetic cost *on average* [22,37,38]. Here, we set [T*, L*] to be the mean stride time and stride length, respectively [16]. Accordingly, we took the cost function to be the expected value of:

$$C=\alpha e^{2}+\beta p^{2}+\gamma u_{1}^{2}+\delta u_{2}^{2}.$$ (9)

The first term, *αe* ^2^, depends on the definition of the goal-level error for the task [15]: i.e., either *e* = *e*_*Spd*_ (Eq 6) for maintaining constant speed, or *e* = *e*_*Pos*_ (Eq 8) for maintaining constant position. Cost functions of the same form can in principle be used to test any other hypothesized task strategy formulated using a different candidate goal function. The second term in Eq (9), *βp* ^2^, penalizes the Euclidean distance, *p*, of the state at each stride from the preferred operating point, [T*, L*] [16]. The costs *e* and *p* are both evaluated at the next stride, *n*+1, whereas the last two effort control terms in Eq (9), involving the control input **u** = [*u*_1_, *u*_2_], are evaluated at stride *n*. The positive constants *α*, *β*, *γ*, and *δ* are weights for the different costs in *C*.

We derived the control inputs *u*_1_ and *u*_2_ by solving a classic quadratic optimal control problem with an equality constraint [16,46]. The controllers were chosen to be optimal with respect to the expected value of Eq (9), $E[C]=\bar{C}$. We further required *u*_1_ and *u*_2_ to be single step, unbiased controllers that sought to drive each respective goal function (either Eq 6 or Eq 8) to zero at each stride. This process yielded optimal control inputs obtained analytically as functions of the current controller state, **x**_*n*_ (see Ref. [16] for additional details). For the speed controller (i.e., for state update Eq 5, with goal function specified by Eq 6), we obtained:

$$\begin{array}{l} u_{1}=\frac{-T_{n}\left[\left(\delta +\sigma_{N2}^{2}\alpha +\left(\sigma_{N2}^{2}+1\right)\beta \right)v^{2}+\beta \right]+L_{n}v\left[\delta +\sigma_{N2}^{2}\left(\alpha +\beta \right)\right]+\beta \left(L^{*}v+T^{*}\right)}{\left[\delta +\left(\sigma_{N1}^{2}+\sigma_{N2}^{2}\right)\alpha +\left(\sigma_{N2}^{2}+1\right)\beta \right]v^{2}+\left(\sigma_{N2}^{2}+1\right)\beta +\gamma }\\ u_{2}=\frac{-L_{n}\left[\gamma +\left(\sigma_{N1}^{2}\alpha +\beta \right)v^{2}+\left(\sigma_{N1}^{2}+1\right)\beta \right]+T_{n}v\left[\gamma +\sigma_{N1}^{2}\left(\alpha v^{2}+\beta \right)\right]+\beta v\left(L^{*}v+T^{*}\right)}{\left[\delta +\left(\sigma_{N1}^{2}+\sigma_{N2}^{2}\right)\alpha +\left(\sigma_{N2}^{2}+1\right)\beta \right]v^{2}+\left(\sigma_{N2}^{2}+1\right)\beta +\gamma } \end{array}$$ (10)

Applying the same procedure to the position controller (i.e., for state update Eq 7, with the goal function specified by Eq 8), the resulting controller inputs have the form *w*_*i*_ = *u*_*i*_ + *ũ*_*i*_, where the *u*_*i*_ are the same controllers derived previously (i.e., Eq 10) but now with the addition of the following terms:

$$\begin{array}{l} {\tilde{u}}_{1}=\frac{\left(\delta +\sigma_{N2}^{2}\alpha +\left(\sigma_{N2}^{2}+1\right)\beta \right)vP_{n}}{\left[\delta +\left(\sigma_{N1}^{2}+\sigma_{N2}^{2}\right)\alpha +\left(\sigma_{N2}^{2}+1\right)\beta \right]v^{2}+\left(\sigma_{N2}^{2}+1\right)\beta +\gamma }\\ {\tilde{u}}_{2}=\frac{-\left(\sigma_{N1}^{2}\alpha v^{2}+\left(\sigma_{N1}^{2}+1\right)\beta +\gamma \right)vP_{n}}{\left[\delta +\left(\sigma_{N1}^{2}+\sigma_{N2}^{2}\right)\alpha +\left(\sigma_{N2}^{2}+1\right)\beta \right]v^{2}+\left(\sigma_{N2}^{2}+1\right)\beta +\gamma } \end{array}$$ (11)

These models illustrate that maintaining speed and position are directly related. Indeed, controlling speed is a *special case* of controlling position. Likewise, because *P*_*n*_ is observable but not directly controllable, controlling position inherently requires that we also control corresponding fluctuations in speed.

### Simulated Walking Data

We generated simulated walking data for two versions of each of the two models (speed control and position control). First, to implement the strictly optimal MIP-based control models (“S_MIP_” and “P_MIP_”), we set *β* = 0 in Eq 9, so the cost function depended only on the goal-level error, *e*, and the effort terms, *u*_1_ and *u*_2_. We then also set the gain matrix in the stride map (Eq 4) to identity, G = I (i.e., *g*_1_ = *g*_2_ = 1 in Eq 5 or 7) [16]. Thus, these strictly optimal MIP controllers optimally corrected for deviations *only* with respect to each specific goal function (Eq 6 or Eq 8) implemented.

Second, we previously found that human walking dynamics were best described by a controller that included a preferred operating point that was also *sub*-optimal in that it slightly *over*-corrected errors with respect to the GEM [16]. To implement these over-correcting control models (“S_OVC_” and “P_OVC_”), we set *β* = 2.79 in Eq (9) and we increase the controller gains to *g*_1_ = *g*_2_ = 1.24 in Eq (5) or (7), respectively [16]. Thus, these sub-optimal controllers tended to correct very strongly (indeed, slightly more than needed) for deviations away from each respective GEM and weakly for deviations along them.

All models were implemented assuming a walking speed of *v* = 1.21 m/s, which corresponded to the mean preferred walking speed of our human subjects (see below). We then chose the remaining parameter values by trial-and-error to approximate the experimentally observed stride speed variability. Thus, *N* and **η** were defined using *σ*_N1_ = *σ*_η1_ = 0.017 and *σ*_N2_ = *σ*_η2_ = 0.010. The remaining weights in the cost function (Eq 9) were set to *α* = *γ* = *δ* = 10. For the sub-optimal control models, we used T* = 1.105 s to correspond to the aggregate mean stride time of all human subjects and L* = vT*, where again *v* = 1.21 m/s. (See [16] for additional details). The primary qualitative aspects of our results were not sensitive to the particular choices of these parameters and no explicit attempts were made to rigorously “fit” our experimental data exactly.

For each of the four model configurations (S_MIP_, P_MIP_, S_OVC_, and P_OVC_), we simulated 20 trials of walking of 1000 strides each to represent a single simulated “average” subject. Model outputs consisted of time series of stride times (*T*_*n*_) and stride lengths (*L*_*n*_). For each simulation, we computed the net cumulative distances walked at each step, to ensure no simulation “walked off” the treadmill [16].

### Experimental Participants

The experimental data used here were drawn from the same data set as analyzed in [16] and are available from Dryad (http://dx.doi.org/10.5061/dryad.sk55m) [47]. Seventeen young healthy adults participated (Table 1). No participants reported any history of orthopedic problems, recent lower extremity injuries, any visible gait anomalies, or were taking medications that may have influenced their gait. Analyses of the variability [24], dynamic stability [31], and stepping dynamics [16] of these participants were reported previously.

**Table 1:** Participant Characteristics.

Gender (M / F)	12 / 5
Age (years)	23.3 ± 2.7
Height (m)	1.73 ± 0.094
Body Mass (kg)	71.1 ± 9.86
Body Mass Index (kg/m^2^)	23.5 ± 1.7

Values shown are mean ± standard deviation.

### Ethics Statement

All procedures were approved by the Institutional Review Board of the University of Texas at Austin. All participants signed approved written informed consent forms prior to participation.

### Experimental Procedures

Subjects walked on a level motor-driven treadmill (Desmo S model, Woodway USA, Waukesha WI) while wearing comfortable walking shoes and a safety harness (Protecta International, Houston TX) that allowed natural arm swing [31]. Preferred self-selected walking speed (PWS) was determined using a standardized protocol [23]. Following a 2-minute rest, subjects completed two 5-minute walking trials at each of five speeds, presented in pseudo-random order. Subjects rested at least 2 minutes between each trial to prevent fatigue. Subjects were instructed to look ahead and avoid extraneous movements while walking. Because differences across walking speeds were described in detail elsewhere [24,31] and were minimal for analyses similar to those conducted here [16], only the data from the preferred walking speeds (PWS) were analyzed here. Data from 1 PWS trial from each of 2 subjects had to be discarded due to poor data quality.

Kinematic data regarding the movements of each foot were recorded at 60 Hz continuously for each entire 5 minute trial using an 8-camera Vicon 612 motion capture system (Oxford Metrics, UK) using methods previously described [24,31]. Raw marker data were then resampled to 1200 Hz to obtain more precise estimates of individual gait events. Stride times (*T*_*n*_) were computed for each stride, *n*, as the time from one heel contact to the next ipsilateral heel contact. Step length was defined as the anterior-posterior distance between the heel and the contralateral heel at each heel contact, when both feet were in contact with the treadmill belt. Stride lengths (*L*_*n*_) were computed as the sum of each pair of 2 consecutive step lengths that composed each stride. The 32 experimental trials analyzed here included an average of 275±17 (mean ± standard deviation) total strides each (range: 242–309).

### Data Analyses

The primary gait variables obtained from each simulated or experimental walking trial consisted of time series of *L*_*n*_ and *T*_*n*_ for all strides within that trial. Time series of stride speeds (*S*_*n*_) and absolute positions (*P*_*n*_) for all strides for each trial were also calculated. Time series of stride speeds were calculated as:

$$S_{n}=L_{n}/T_{n}.$$ (12)

For each subject, average walking speed was computed as the average stride speed, $v=\bar{S}$, computed across all *N* strides within each trial. Absolute positions relative to the treadmill were computed for each stride by summing the stride-to-stride displacements over all previous consecutive strides:

$$P_{n}=\sum_{i=1}^{n}\left(L_{i}-vT_{i}\right).$$ (13)

All analyses were then applied to these four time series for each simulated and experimental trial.

First, we computed the mean and standard deviation of each time series (*L*_*n*_, *T*_*n*_, *S*_*n*_, and *P*_*n*_). Of primary interest were the dynamics of those fluctuations perpendicular to each respective GEM (e.g., the “*δ*_*P*_” deviations indicated in Fig 1B). For any GEM, the corresponding *δ*_*P*_ deviations are directly “goal relevant” precisely because they indicate errors with respect to the defined task goal. Here, for the constant speed task, fluctuations in *S*_*n*_ directly reflect *δ*_*P*_ deviations away from the speed GEM (Fig 1B; [16]). For the constant position task, fluctuations in *P*_*n*_ directly reflect *δ*_*P*_ deviations away from the position GEM.

Standard deviations only quantify the average magnitude of differences across all strides, regardless of temporal order, and thus yield no information about how each stride affects subsequent strides. Therefore, Detrended Fluctuation Analysis (DFA) [32,48,49] was used to define a lag-independent measure of statistical persistence across successive strides in each time series [16,34]. In brief, DFA calculates a scaling exponent *α*. Values of *α* > ½ indicate *persistence* [32,48]: deviations in one direction are more likely to be followed by deviations in the same direction. Values of *α* < ½ indicate *anti*-persistence: deviations in one direction are more likely to be followed by deviations in the opposite direction. A value of *α* = ½ indicates uncorrelated (i.e., white) noise. A value of *α* = 1½ indicates brown noise (i.e., integrated white noise) [32,48], equivalent to Brownian motion. In the context of control, variables *not* tightly regulated [16,34] typically exhibit stronger statistical persistence (*α* >> ½), whereas more tightly regulated variables typically exhibit fluctuations with *α* ≈ ½ [16,34]. Here, we predicted that trying to maintain approximately constant *speed* would yield *α*(*S*_*n*_) very close to ½ and *α*(*P*_*n*_) very close to 1½ (since displacement is the integral of velocity). Conversely, we predicted that trying to maintain approximately constant *position* would yield *α*(*P*_*n*_) very close to ½ and hence *α*(*S*_*n*_) << ½. Lastly, we predicted humans would exhibit fluctuation dynamics consistent with speed control [16], but contradictory to position control, even though both strategies predict the same overall *average* behavior.

However, DFA analyses only capture the degree to which, on average, deviations were corrected in *time* (i.e., from stride-to-stride). They do not provide explicit information regarding how fluctuations depend on the controller *state*. A controller trying to maintain some constant average position, $\bar{P}$, on the treadmill (Eq 3), when observing any deviation away from $\bar{P}$ on a given stride (i.e., $P_{n}^{\prime }=P_{n}-\bar{P}$), should correct that deviation on the subsequent stride by making a corresponding change in position, Δ*P*_*n*+1_ = *P*_*n*+1_ − *P*_*n*_, in the opposite direction. Likewise, a controller trying to maintain some constant average speed, $v=\bar{S}$ (Eq 2), when observing any deviation $S_{n}^{\prime }=S_{n}-\bar{S}$, should correct that deviation by a subsequent change in speed, Δ*S*_*n*+1_ = *S*_*n*+1_ − *S*_*n*_, in the opposite direction. We therefore constructed, for each controller and for our human subjects, plots of Δ*P*_*n*+1_ vs. $P_{n}^{\prime }$ and Δ*S*_*n*+1_ vs. $S_{n}^{\prime }$, and computed the linear slopes (using least-squares regression) and strength of correlation (r^2^) for each corresponding relationship. We predicted that position control would yield slopes close to −1 for Δ*P*_*n*+1_ vs. $P_{n}^{\prime }$ with high correlation, whereas speed control would yield slopes close to −1 for Δ*S*_*n*+1_ vs. $S_{n}^{\prime }$ with high correlation, but slopes close to 0 with very low correlation for Δ*P*_*n*+1_ vs. $P_{n}^{\prime }$. We predicted humans would exhibit stepping corrections consistent with speed control [16], but contradictory to position control.

## Results

**Fig 2:**
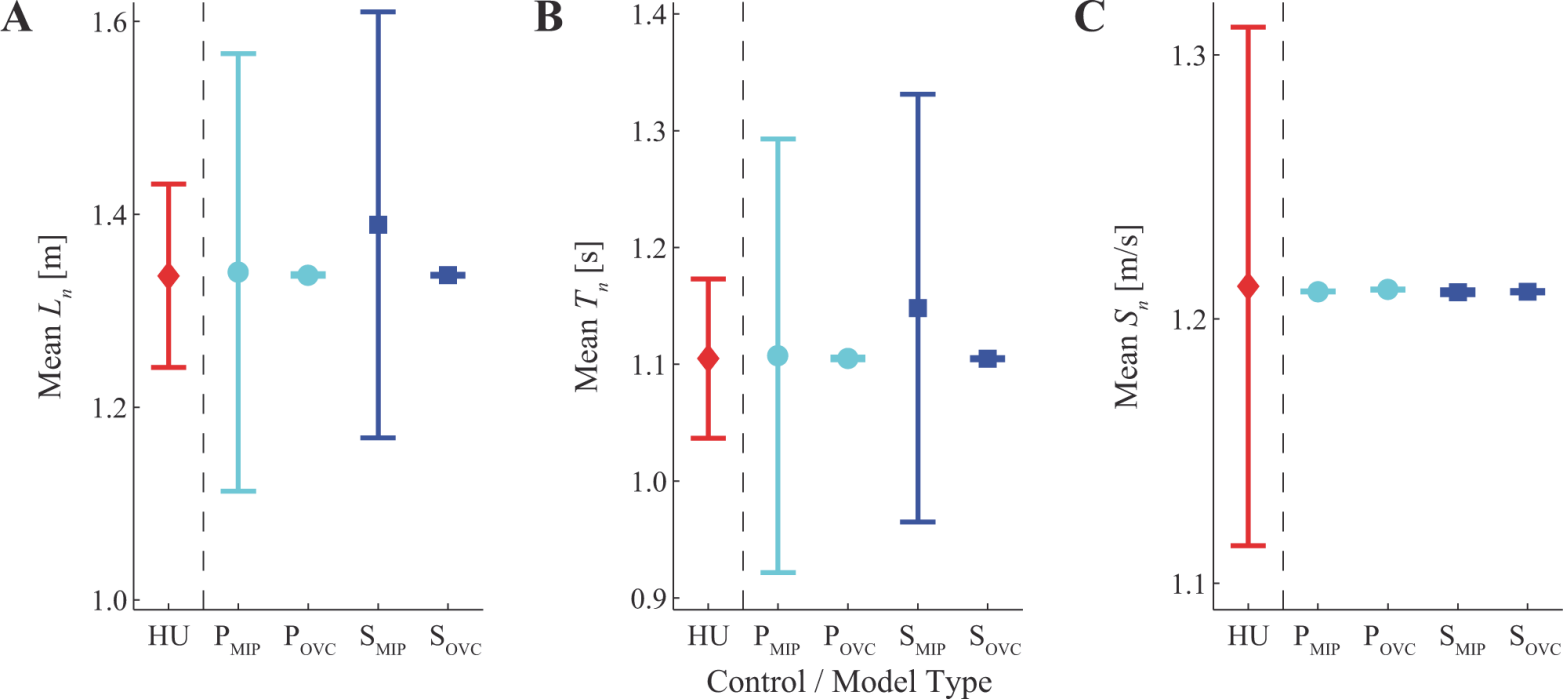
Means of Basic Gait Variables. (A) Stride Lengths (*L*_*n*_), (B) Stride Times (*T*_*n*_), and (C) Stride Speeds (*S*_*n*_ = *L*_*n*_/*T*_*n*_). In each sub-plot, data shown are for healthy human subjects (HU), the Position Control model with MIP control (P_MIP_), the Position Control model with “over-correcting” control (P_OVC_), the Speed Control model with MIP control (S_MIP_), and the Speed Control model with “over-correcting” control (S_OVC_). For the HU data, error bars indicate between-subject ± standard deviations. For the model data, error bars indicate between-trial ± standard deviations. All models yielded mean stride parameters well within the experimental range. However, both MIP controllers (P_MIP_ and S_MIP_) yielded much greater between-trial variance for both *L*_*n*_ (A) and *T*_*n*_ (B) than observed experimentally.

On average, all four model formulations predicted mean stride behavior essentially indistinguishable from that of humans (Fig 2). By construction, mean stride speeds (*S*_*n*_) for each model matched the mean of the experimental results (Fig 2C). For the sub-optimal over-correcting models (P_OVC_ and S_OVC_), this also yielded very consistent mean stride lengths (*L*_*n*_; Fig 2A) and stride times (*T*_*n*_; Fig 2B). The pure MIP models (P_MIP_ and S_MIP_) exhibited much greater between-trial variance in the mean values of *L*_*n*_ and *T*_*n*_. This occurred because there was no “preferred” operating point defined (i.e., *β* = 0 in Eq 9) and therefore no cost associated with moving away from any particular operating point along the GEM. Fluctuations in *P*_*n*_ were already normalized to each trial’s own mean position, so mean *P*_*n*_ values (not shown) were all zero by construction.

**Fig 3:**
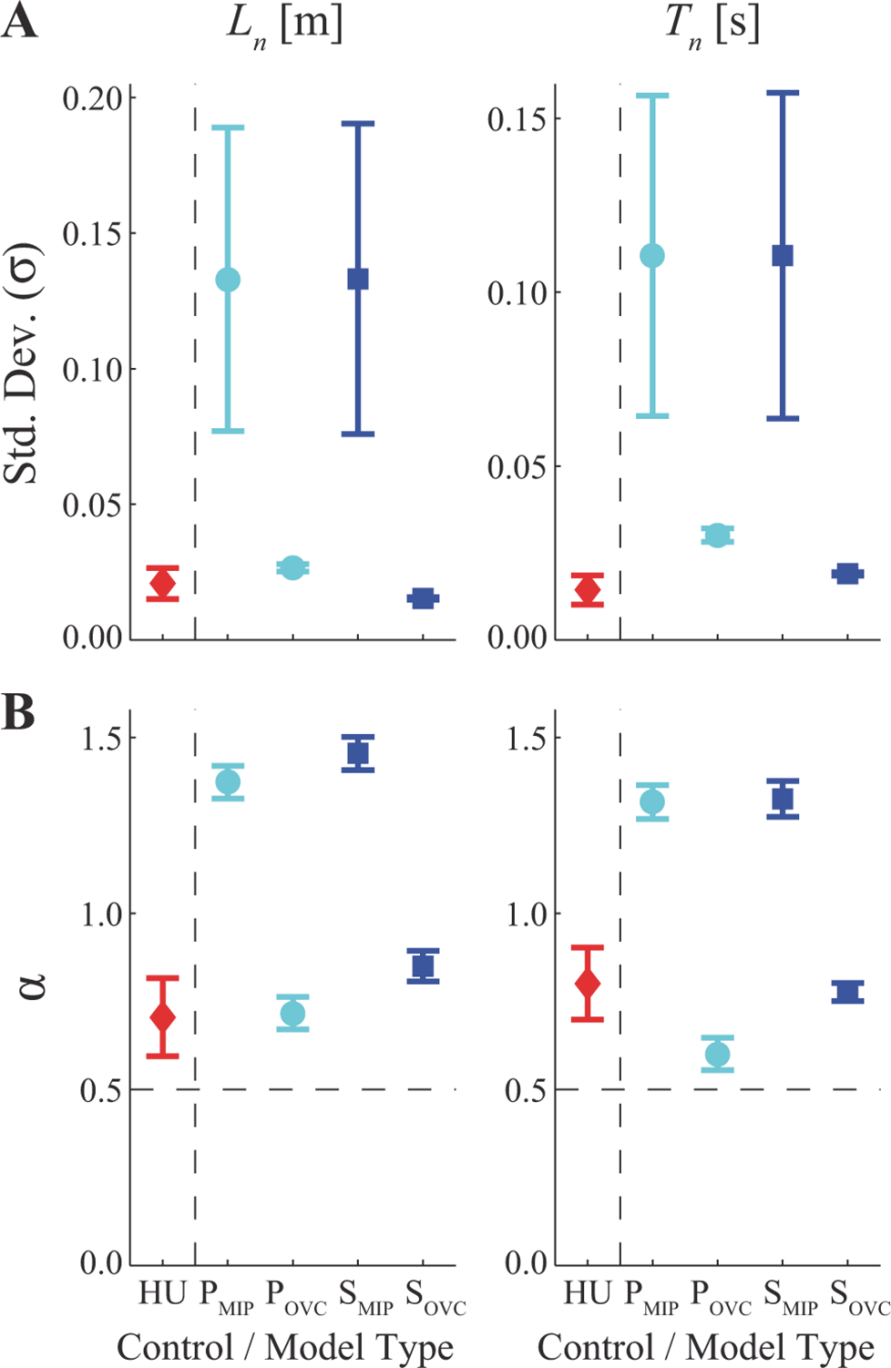
Variability (σ) and Statistical Persistence (α) of Stride Lengths (*L*_*n*_) and Stride Times (*T*_*n*_). (A) Variability (within-trial standard deviations (σ) of humans (HU) and of each of the four model configurations (P_MIP_, P_OVC_, S_MIP_, and S_OVC_). (B) DFA scaling exponents (α) of humans (HU) and of each of the four model configurations (P_MIP_, P_OVC_, S_MIP_, and S_OVC_). For the HU data, error bars indicate between-subject ± standard deviations. For the model data, error bars indicate between-trial ± standard deviations. Both models that implemented pure MIP-type control (i.e., P_MIP_ and S_MIP_), regardless of whether controlling for speed or position, yielded much higher levels of variance (A) and much greater degrees of statistical persistence (indeed, approaching random walk behavior: α ≈ 1.5) (B) in the typical walking parameters that are not directly controlled for (i.e., *L*_*n*_ and *T*_*n*_) than did humans (HU).

Both pure MIP models (P_MIP_ and S_MIP_) also predicted much greater within-trial stride-to-stride variability (Fig 3A) and statistical persistence (Fig 3B) for both *L*_*n*_ and *T*_*n*_ than humans exhibited. Both pure MIP control models were thus substantially qualitatively different from humans. Conversely, in terms of *L*_*n*_ and *T*_*n*_, both sub-optimal models, whether enacting speed control (S_OVC_) or position control (P_OVC_), predicted both the variances (Fig 3A) and statistical persistence (Fig 3B) exhibited by humans equally well.

**Fig 4:**
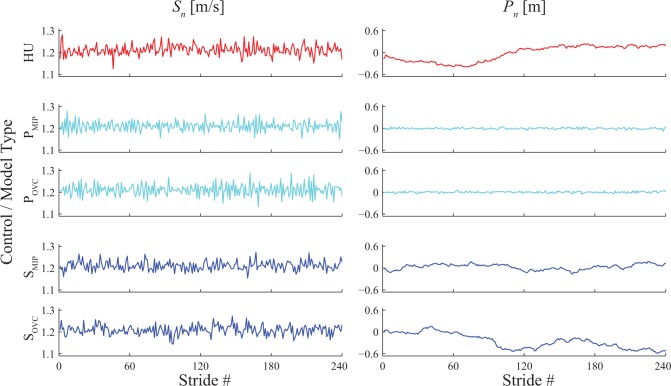
Example Time Series of Stride Speeds (*S*_*n*_) and Absolute Position (*P*_*n*_). Data are shown for 240 consecutive strides from a typical trial for a typical human subject (HU) and for one typical trial each of each of the four model configurations (P_MIP_, P_OVC_, S_MIP_, and S_OVC_). All four model configurations yielded *S*_*n*_ time series that initially appeared qualitatively similar both to each other and also to humans (HU). Conversely, both of the Position Control models (P_MIP_ and P_OVC_) qualitatively exhibited far less variance and far less “drift” in their absolute positions on the treadmill (*P*_*n*_) than did humans (HU).

Qualitatively, all four model formulations exhibited fluctuations in stride speeds (*S*_*n*_) that appeared largely similar to those of humans (Fig 4). However, fluctuations in absolute positions (*P*_*n*_) for both humans and for each *speed* control model (S_MIP_ and S_OVC_) exhibited large-amplitude, low-frequency behavior, consistent with subjects “drifting” forward and backward on the treadmill over time [16]. Conversely, fluctuations in *P*_*n*_ for both *position* control models (P_MIP_ and P_OVC_) were very narrowly centered near *P*_*n*_ = 0 (Fig 4).

**Fig 5:**
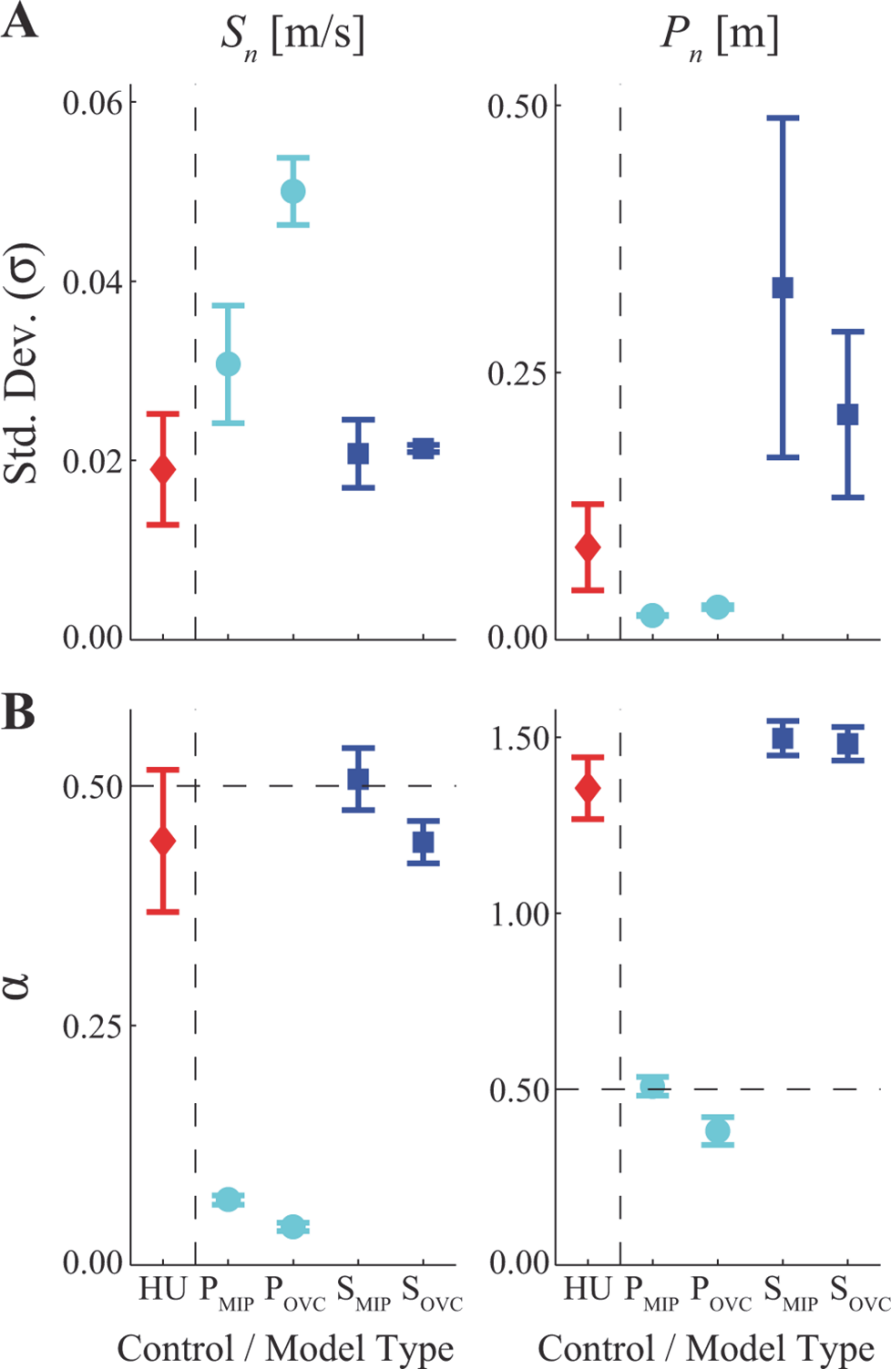
Variability (σ) and Statistical Persistence (α) of Stride Speeds (*S*_*n*_) and Absolute Positions (*P*_*n*_). (A) Variability (within-trial standard deviations (σ) of humans (HU) and of each of the four model configurations (P_MIP_, P_OVC_, S_MIP_, and S_OVC_). (B) DFA scaling exponents (α) of humans (HU) and of each of the four model configurations (P_MIP_, P_OVC_, S_MIP_, and S_OVC_). For the HU data, error bars indicate between-subject ± standard deviations. For the model data, error bars indicate between-trial ± standard deviations. Position control yielded uncorrelated fluctuations (α ≈ 0.5) of absolute positions (*P*_*n*_) under optimal MIP control conditions (P_MIP_) and slightly anti-correlated fluctuations (α < 0.5) of *P*_*n*_ when over-correcting for position (P_OVC_). This in turn yielded *highly* anti-correlated fluctuations (α < 0.1) of stride speeds (*S*_*n*_). Speed control yielded uncorrelated fluctuations (α ≈ 0.5) of stride speeds (*S*_*n*_) under optimal MIP control conditions (S_MIP_) and slightly anti-correlated fluctuations (α < 0.5) of *S*_*n*_ when over-correcting for position (S_OVC_). This in turn yielded strongly correlated fluctuations for absolute position (*P*_*n*_), which approached random walk behavior: α ≈ 1.5), consistent with position reflecting integrated speed (Eq 1, 3, and 13). Both of the position control models (P_MIP_ and P_OVC_) exhibited fluctuation dynamics *very* different from those of humans (HU). The over-correcting speed control model (S_OVC_) exhibited fluctuation dynamics most consistent with humans.

These qualitative observations were confirmed by our analyses (Fig 5). Both *position* control models (P_MIP_ and P_OVC_) exhibited slightly greater variability for *S*_*n*_, but far less variability for *P*_*n*_, than did humans (Fig 5A). Conversely, the *speed* control models (S_MIP_ and S_OVC_) exhibited variability very similar to humans for *S*_*n*_, but greater variability for *P*_*n*_ (Fig 5A). Most importantly, however, both *speed* control models (S_MIP_ and S_OVC_) exhibited fluctuations in stride speed (*S*_*n*_) that were either very near to, or slightly less than, *α* = ½, respectively (Fig 5B). This then yielded fluctuations in absolute position (*P*_*n*_) that closely approximated Brownian motion (Fig 5B), as predicted. Conversely, both *position* control models (P_MIP_ and P_OVC_) exhibited fluctuations in absolute position (*P*_*n*_) that were either very near to, or slightly less than, *α* = ½, respectively (Fig 5B). This then yielded fluctuations in speed (*S*_*n*_) that exhibited extremely small *α* << ½ (Fig 5B). The fluctuation dynamics for *S*_*n*_ and *P*_*n*_ for both of the two *speed* control models closely approximated those of humans (Fig 5B). However, both *position* control models were qualitatively very different from humans.

**Fig 6:**
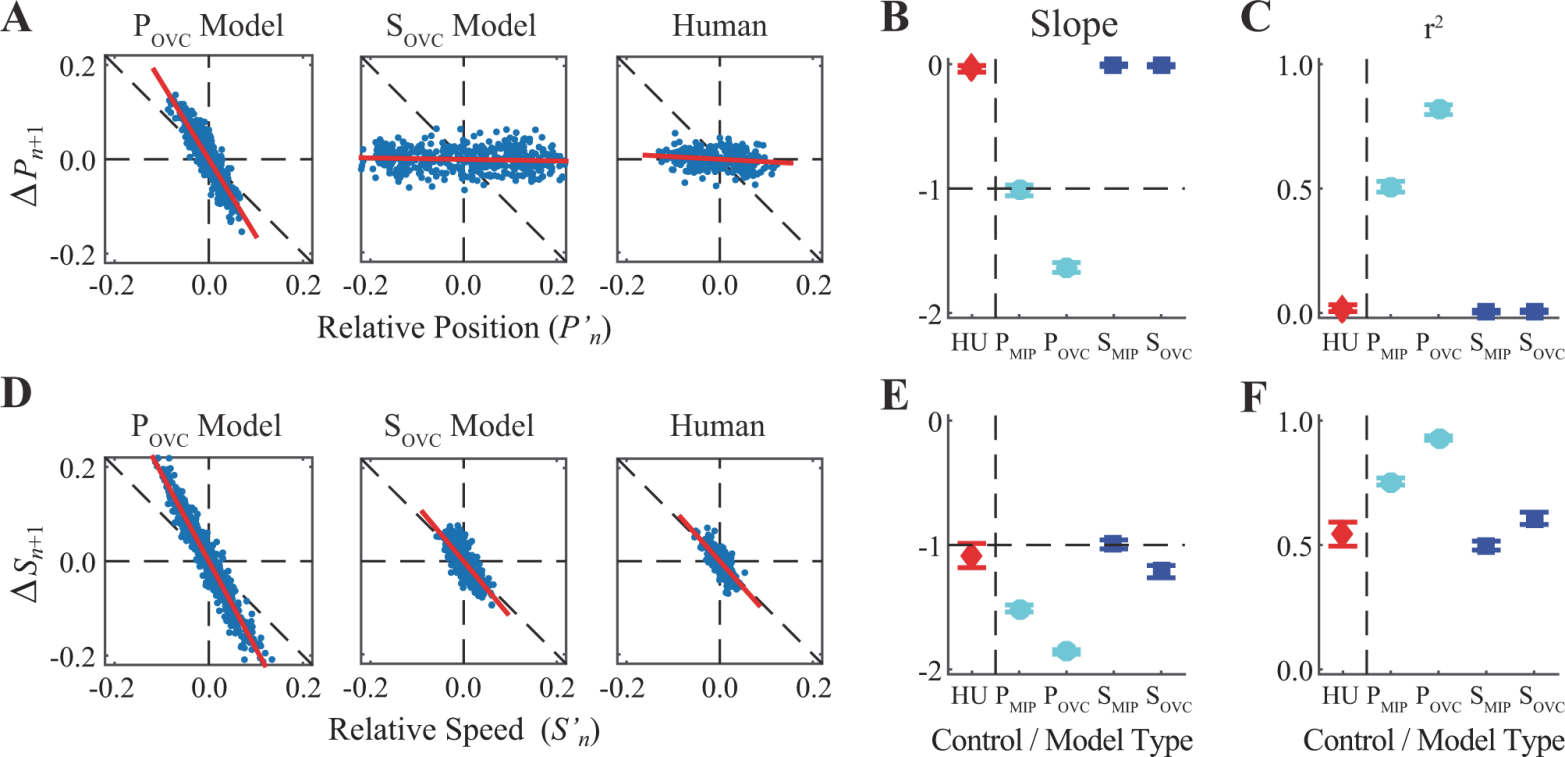
Direct Correction of Errors in Position and Speed. (A) Exemplary plots of how errors in relative position $\left(P_{n}^{\prime }=P_{n}-\bar{P}\right)$ were corrected on each subsequent stride (Δ*P*_*n+1*_ = *P*_*n+1*_ − *P*_*n*_). Data are shown from 1 trial each for each of the two sub-optimal control model configurations (P_OVC_ and S_OVC_) and for 1 typical human subject. Diagonal lines indicate least-squares fits to each data set. “Perfect” error correction would yield a relationship with a slope of −1 and a strong correlation. (B) Summary results for the slopes of these relationships for all trials for both humans (HU) and for each of the four model configurations (P_MIP_, P_OVC_, S_MIP_, and S_OVC_). For the HU data, error bars indicate between-subject ± standard deviations. For the model data, error bars indicate between-trial ± standard deviations. (C) Summary results for the strength of correlation (r^2^) of these relationships [error bars are defined in the same manner as in part (B)]. While both position control models (P_MIP_ and P_OVC_) exhibited steep slopes (B) with strong correlations (C), both humans and both speed control models (S_MIP_ and S_OVC_) exhibited nearly zero slopes with extremely weak correlations, consistent with *not* correcting deviations in position. (D) Exemplary plots of how errors in relative speed $\left(S_{n}^{\prime }=S_{n}-\bar{S}\right)$ were corrected on each subsequent stride (Δ*S*_*n+1*_ = *S*_*n+1*_ − *S*_*n*_). Data are shown in the same manner as in (A). As in (A), “perfect” error correction would yield a relationship with a slope of −1 and a strong correlation. (E) Summary results for the slopes of these relationships for all trials for both humans (HU) and for each of the four model configurations (P_MIP_, P_OVC_, S_MIP_, and S_OVC_) [error bars are defined in the same manner as in part (B)]. (F) Summary results for the strength of correlation (r^2^) of these relationships [error bars are defined in the same manner as in part (B)]. Human subjects exhibited nearly perfect corrections (i.e., slope ≈ −1: (E)) for errors in speed with strong correlations (F), as did both of the speed control models (S_MIP_ and S_OVC_).

Both *position* control models corrected deviations in absolute position, $P_{n}^{\prime }$, by enacting opposing changes in position, Δ*P*_*n*+1_, on subsequent strides (Fig 6A). These models predicted slopes of Δ*P*_*n*+1_ vs. $P_{n}^{\prime }$ of ~ −1 for optimal (P_MIP_) and < −1 for sub-optimal (P_OVC_) control (Fig 6B), with both exhibiting strong correlations (Fig 6C). As expected, both *position* control models also exhibited strong over-corrections for changes in speed (Fig 6D–6F). Conversely, the *speed* control models predicted slopes of Δ*S*_*n*+1_ vs. $S_{n}^{\prime }$ of ~ −1 for optimal (S_MIP_; Fig 6D–6E) and slightly < −1 for sub-optimal (S_OVC_) control (Fig 6E), with both exhibiting strong correlations (Fig 6F). These *speed* control models, however, predicted essentially no corrections for deviations in relative position (Fig 6A–6C). Humans exhibited slopes of slightly < −1 for Δ*S*_*n*+1_ vs. $S_{n}^{\prime }$ (Fig 6D–6E) with strong correlations (Fig 6F), but slopes of ~ 0 for Δ*P*_*n*+1_ vs. $P_{n}^{\prime }$ (Fig 6A–6B), with nearly zero correlations (Fig 6C). These experimental results were highly consistent with predictions of the *speed* control models, but substantially qualitatively different from predictions of either *position* control model.

## Discussion

Increased locomotor variability can lead to detrimental consequences like increased energetic cost [50] or increased fall risk [10]. Conversely, exploiting redundancy can facilitate motor performance [19,28] and may help explain why encouraging variability accelerates improvements in robot-assisted gait re-training [11,12]. Here, we adopted a model-based approach that allowed us to test concrete *a priori* hypotheses [14,16,20] to determine how humans regulate sagittal plane stepping movements as they walk on a motor-driven treadmill. We defined explicit goal functions [15,20] to compare two candidate control strategies: i.e., regulating stride-to-stride *speed* vs. absolute *position*. Both strategies are equally plausible and yielded average behavior indistinguishable both from each other and from humans (Fig 2). However, these strategies predicted *very* different patterns of stride-to-stride fluctuation dynamics in the relevant variables (Figs 3–6). Equivalent analyses of experimental data yielded results well-predicted by the speed-control model (S_OVC_), but not by either position-control model (P_MIP_ or P_OVC_). These simulation results are consistent with the notion that humans do not try to stay in the same absolute *position* on the treadmill at each stride, but instead try, at least primarily, to maintain the same *speed* as the treadmill. This work directly extends a computational framework developed for studying trial-to-trial fluctuation dynamics in repetitive human movements [15,20], including walking [16]. Our method adapts approaches independently developed using time series analyses [30,48], redundancy [15], and feedback control [18] and unifies these ideas under a coherent theoretical framework [20]. In contrast to approaches that seek to identify single “optimal” average solutions [21,22], or that only analyze “variability” [7,14], the present results clearly demonstrate that one must account for trial-to-trial (here, stride-to-stride) fluctuation dynamics [28,29,51] to identify appropriate control policies.

In theory, there are numerous ways humans might control their stepping movements, since any strategy that satisfies Eq. (1) could be allowed. Several plausible alternatives beyond those presented here were previously discussed in [16]. Yet another alternative the present work suggests might be to adopt “lazy” position control. Participants could allow their position to drift uncorrected anywhere within the middle of the treadmill belt and *only* correct position errors when they walked “too close” to either end of the treadmill belt (e.g., Fig 1A). Effectively: if you are too far forward, move back; if you are too far back, move forward. Indeed, such a strategy, a form of deadband control for position, would exemplify the Minimum Intervention Principle [14,18]. The analyses in Fig 6 address this possibility. Humans adopting such a strategy would be expected to take shorter and/or slower strides at more forward positions (i.e. at large positive values of relative position, *P′*_*n*_) to enact larger *negative* changes in position (Δ*P*_*n*+1_). At more backward positions (i.e. at large negative values of *P′*_*n*_), participants would be expected to take longer and/or faster strides to enact larger *positive* Δ*P*_*n*+1_. For intermediate values of *P′*_*n*_, with such a deadband controller, participants would be expected to exhibit little or no correction for position errors (i.e., Δ*P*_*n*+1_ ≈ 0). Together, the overall result would be a distribution of data points, unlike those in Fig 6A, that would be flat in the middle, but would curve sharply up on the left and sharply down on the right. However, no such nonlinearity was observed (Fig 6A; Human). Indeed, human subjects exhibited a relationship between *P′*_*n*_ and Δ*P*_*n*+1_ that was in fact quite flat (i.e., Δ*P*_*n*+1_ ≈ 0) across the entire range of positions explored by all subjects tested.

Similarly, Wang and Srinivasan [52] used experimental data of current foot and pelvis states to predict subsequent anterior and lateral foot placements. Adding current position on the treadmill as an additional predictor in their regression models explained little to no (< 5%) additional variance in foot placement. They also concluded that healthy humans do not try to maintain absolute position on the treadmill (what they called “station-keeping”). Their participants walked on treadmills shorter (1.27 or 1.52 m long) than that used here (1.73 m long), demonstrating that healthy humans do not tightly regulate absolute position even when walking on shorter treadmills. Thus, while such an intermittent, boundary-driven, deadband position control policy might be a very rational and simple strategy to adopt, healthy humans do not use this type of control.

Likewise, the models derived here all employed single-stride direct error feedback control. However, there is no physiological necessity to fully correct movement errors over only a single stride. Another less restrictive approach would be to correct errors more slowly over multiple strides. Indeed, some have derived control policies for robots that regulate walking over many consecutive steps [53,54]. However, our purpose here was to identify the *simplest possible* model(s) that could capture the *primary* features of inter-stride fluctuations observed in our experiments. We therefore chose to start with *k*-step optimal controllers and our review of the literature suggested *k* = 1 should provide a sufficient first approximation. Indeed, mechanically stable walking can be maintained by implementing control only one step at a time [55,56]. Physiologically, the intrinsic timescales of walking (i.e., roughly 1000–1200 ms/stride ≈ 500–600 ms/step; Fig 2B) allow ample time for even slow reflex responses [4] to operate. In humans, single-step control strategies predict subsequent foot placements during both stepping [57,58] and walking [52,59] quite well. When humans are released from a forward lean [60] or perturbed while walking [61,62], they recover in ≤ 3 steps (i.e., ≤ 1½ strides) and can also modify their stepping responses within a single step (½ stride) [63]. Additionally, visual input is known to be essential for regulating walking [3,64–66]. In multiple experiments where humans were asked to change direction [67,68], step onto specific targets [69,70], avoid obstacles in their path [64,71], walk across complex terrains [65,72,73], or navigate environments with large numbers of densely packed visual targets/obstacles [74,75], visual information about these targets, obstacles, and/or environments was acquired only 1–3 steps (i.e., ½-1½ strides) in advance. Regulating movements only a few steps at a time in this way is consistent with the fact that humans must navigate noisy environments using a noisy neuromotor system [8,27] that must contend with multiple forms and levels of uncertainty [76]. Such a situation makes predicting motor outcomes farther into the future impractical. The simple and parsimonious single-stride control models presented here adequately captured the primary fluctuation dynamics observed in our human subjects during unperturbed walking, consistent with this large body of experimental evidence. While the inter-stride dynamics of human walking may exhibit some features that, in some contexts, would require *multi*-stride controllers to explain, exploration of such models is left to future work.

The present findings have several important implications. First, increased walking variability has been broadly implicated in predicting increased risk of falling in the elderly [9,10]. However, there remains considerable debate as to which gait measures should be included [77]. The confusion on this very important topic likely arises from the absence of a clear framework for understanding where the variability in different gait measures comes from and what its underlying purpose is. Variability arising from uncontrolled noise could be detrimental [6,78], whereas variability that reflects corrective control responses and/or exploration of alternative viable movement strategies is likely beneficial [16,29,79]. The approach presented here provides a theoretical, analytical, and experimental framework within which these critical distinctions can be tested. By determining the appropriate goal functions for efficient and stable walking, we can better identify which candidate body-state variables should be targeted for developing effective therapeutic interventions. As a specific example, developing more rigorous and less heuristic approaches to identifying relevant goal functions could lead to more efficient implementations of so-called “assist-as-needed” or “patient-cooperative” robotic gait retraining programs [12,13,80]. Similarly, such approaches could also be used to guide development of minimally complex control algorithms (e.g., [78,81,82]) that can allow bipedal robots to navigate more complex terrains [5,6] far more efficiently.

The present work focused specifically on the issue of how stepping movements are controlled *between*-strides. This work itself does not address the question of how control is organized at a neurophysiological and/or biomechanical level to achieve the *within*-stride generation of each stepping movement [16]. In this sense, while our work does provide a low-dimensional “template” for identifying features critical to between-stride control, these template control strategies still need to be “anchored” [83,84] within more elaborate, higher-dimensional biological models to identify the underlying neural and/or biomechanical mechanisms through which these control processes are enacted. Likewise, the present work only addresses how stepping movements are controlled in the sagittal plane. Both bipedal walking robots [78,81] and humans [2] are inherently more unstable in the mediolateral direction. While the present work does not address this question, the framework developed here could be extended to also account for lateral stepping movements. Finally, both speed control and position control very easily satisfied the basic requirement (Eq 1) of staying on the treadmill. However, the present results (Figs 3–5) make it clear that, of the types of models tested, human treadmill walking behavior is far more consistent with adopting a strategy that strongly prioritizes speed control over position control. Nevertheless, these results by themselves do not directly answer the question of *why* humans chose to do this or *how* such control is implemented physiologically. Answering these important questions will require a combination of additional more focused experiments and/or developing more strongly “anchored” [83,84] biological models.

It is also important to point out that although we used a stochastic optimal control framework as a convenient computational means to construct our control models, this approach is not unique. We could alternatively have chosen to build equivalent controller models using neural networks, or genetic algorithms, or some other approach (although, it must be added, at the cost of substantially increased model complexity). Additionally, and more importantly, one must not conflate the *methods* used to construct these models with the underlying biological *phenomena* being studied. We do not claim that because our models capture the primary aspects of our experimental data, that the human brain solves the same stochastic optimal control problem or solves it in the same way. It almost certainly does not. However, whatever physiological mechanisms are at play to produce the end result (i.e., our experimental data), that end result must be at least consistent with the models constructed here. Identifying the *physiological* mechanisms used to enact these control policies should evolve as part of the process of developing those more strongly “anchored” [83,84] biological models.

Likewise, the present work focused specifically on identifying how stepping movements are regulated during *treadmill* walking. This raises the question of what these results imply about *overground* walking. Indeed, there has been much debate over the years about how treadmill and overground walking are related. In theory, for an idealized treadmill (i.e. rigid walking surface and constant belt speed), there are no fundamental mechanical differences between the two tasks [85]. However, while several studies reported significant differences [86–89], others have reported minimal or no differences between the two tasks for a range of relevant dependent measures [86,90–92]. It is tempting then to think that perhaps the present findings, showing that healthy humans strongly regulate stride speed during treadmill walking, suggest that humans likely also naturally regulate walking speed in the same way during overground walking. However, this cannot be the case. Indeed, walking speed varies quite considerably over multiple time scales during overground walking [93]. More importantly, while stride-to-stride fluctuations in stride speeds (*S*_*n*_) exhibit slight anti-persistence during treadmill walking (Fig 5B) and while our models confirm this to be a direct result of trying to maintain approximately constant speed across strides, experimental data from overground walking exhibit very strong statistical persistence for *S*_*n*_ [35] that is quite different from treadmill walking. These findings suggest that human locomotor control is very flexible and adaptable to changing task conditions and task demands. The present work demonstrates that a comprehensive model-based and fundamentally dynamical approach, based on testing experimental data against established theoretical predictions, is needed to delineate the differences in stepping control between different locomotor tasks.
